# Doping and temperature evolutions of optical response of Sr_3_(Ir_1-*x*_Ru_*x*_)_2_O_7_

**DOI:** 10.1038/s41598-020-79263-5

**Published:** 2020-12-18

**Authors:** Gihyeon Ahn, J. L. Schmehr, Z. Porter, S. D. Wilson, S. J. Moon

**Affiliations:** 1 Department of Physics, Hanyang University, Seoul, 04763 Republic of Korea; 2 Materials Department, University of California, Santa Barbara, CA 93106 USA; 3 Research Institute of Natural Science, Hanyang University, Seoul, 04763 Republic of Korea

**Keywords:** Condensed-matter physics, Electronic properties and materials

## Abstract

We report on optical spectroscopic study of the Sr_3_(Ir_1-*x*_Ru_*x*_)_2_O_7_ system over a wide doping regime. We find that the changes in the electronic structure occur in the limited range of the concentration of Ru ions where the insulator–metal transition occurs. In the insulating regime, the electronic structure associated with the effective total angular momentum *J*_eff_ = 1/2 Mott state remains robust against Ru doping, indicating the localization of the doped holes. Upon entering the metallic regime, the Mott gap collapses and the Drude-like peak with strange metallic character appears. The evolution of the electronic structure registered in the optical data can be explained in terms of a percolative insulator–metal transition. The phonon spectra display anomalous doping evolution of the lineshapes. While the phonon modes of the compounds deep in the insulating and metallic regimes are almost symmetric, those of the semiconducting compound with *x* = 0.34 in close proximity to the doping-driven insulator–metal transition show a pronounced asymmetry. The temperature evolution of the phonon modes of the *x* = 0.34 compound reveals the asymmetry is enhanced in the antiferromagnetic state. We discuss roles of the *S* = 1 spins of the Ru ions and charge excitations for the conspicuous lineshape asymmetry of the *x* = 0.34 compound.

## Introduction

The dynamics of doped charge carriers in correlated insulators and their interplay with spin and lattice degrees of freedom have been one of the major focuses of condensed matter physics^1,2^. Copper oxides are one of the most outstanding examples, where carrier doping results in various exotic phases^3^, such as pseudogap, spin/charge density wave, and superconducting states. As a 5*d* counterpart of the cuprates, layered perovskite iridium oxides Sr_*n*+1_Ir_*n*_O_3*n*+1_ (*n* = 1 and 2) have attracted recent interest. In Sr_*n*+1_Ir_*n*_O_3*n*+1_, the moderate Coulomb interaction and the strong spin–orbit coupling yield an effective total angular momentum *J*_eff_ = 1/2 Mott state^4–7^. Doping of charge carriers into the single-layered Sr_2_IrO_4_ indeed leads to a number of anomalous phenomena, paralleling those of the cuprates. Angle-resolved photoemission studies of the electron-doped Sr_2_IrO_4_ report the observation of the pseudogap^8,9^ and *d*-wave gap^10^. Scanning tunneling spectroscopy (STS) measurements also detect the signatures of the pseudogap^11,12^ and *d*-wave gap^12^. Further a manifestation of the unidirectional order is registered in a STS^11^ and a neutron scattering^13^ experiments.

The bilayer iridate Sr_3_Ir_2_O_7_ is more susceptible to doping-induced metallization than Sr_2_IrO_4_, because its charge gap is smaller^6,14,15^. Indeed a slight electron doping via La substitution is found to induce an abrupt insulator–metal transition leading to a homogeneous correlated metallic state in (Sr_1-*y*_La_*y*_)_3_Ir_2_O_7_ with *y* > 0.04^16^, while the inhomogeneity persists in the metallic (Sr_1-*y*_La_*y*_)_2_IrO_4_ up to the highest La-substitution levels of *y* ≈ 0.06^17^. Apart from this difference, they share common phenomenology of the unidirectional order. STS^18^ and polarized Raman spectroscopy^19^ measurements on (Sr_1-*y*_La_*y*_)_3_Ir_2_O_7_ show that a unidirectional order with the same symmetry and ordering vector as the structural distortion^16,20^ appears upon electron doping. A charge-density-wave-like order was also reported in an ultrafast optical reflectivity experiment of La-doped Sr_3_Ir_2_O_7_ which shows development of a coherent amplitude oscillations of an electronic order parameter^21^.

The *B*-site substitution in the bilayer iridates leads to the phenomena quite distinct from those observed in the *A*-site substituted ones_._ While the La substitution leads to a homogeneous metallic state^16^, Ru substitution gives rise to nanometer-scale phase separation across a wide doping range^22,23^. Upon Ru substitution, the ground state evolves from an antiferromagnetic insulator to a paramagnetic metal (*x* > 0.70) via an intervening antiferromagnetic metal (0.35 < *x* < 0.70)^22,23^. STS measurements of Sr_3_(Ir_1-*x*_Ru_*x*_)_2_O_7_ with 0.0 ≤ *x* ≤ 0.50 show that the doped holes remain localized over nanometer-length scales at low Ru concentrations and an insulator–metal transition occurs at a critical concentration of *x*_c_ = 0.35^22^. The nanoscale spatial inhomogeneity is observed even in the fully metallic and antiferromagnetic compound of *x* = 0.50. The STS data of this compound show V-shaped gap which is attributed to the effects of disorder^23^. Combined with the magnetization and neutron scattering measurements, it is suggested that the interplay between localized and itinerant regions may stabilize the antiferromagnetic metallic phase^22^. Despite these observations, little is known on the dynamics of doped charge carriers and on the interplay among charge, spin, and lattice degrees of freedom in the Sr_3_(Ir_1-*x*_Ru_*x*_)_2_O_7_ system. Optical spectroscopy is ideally suited to address this issue.

In this paper, we report on optical spectroscopy study of the Sr_3_(Ir_1-*x*_Ru_*x*_)_2_O_7_ system in a wide doping region covering the phase diagram. We investigate doping evolution of the electronic response and phonon dynamics. We find that the low-temperature optical spectra reflecting the electronic structure of the ground states show a noticeable change with Ru substitution only in the *x* region close to the critical concentration *x*_c_ = 0.35 at which the insulator–metal transition occurs^22^. In the insulating regime with low Ru concentrations, the optical conductivity spectra exhibit the optical excitations across the Mott gap between the *J*_eff_ = 1/2 bands at 0.4 eV, which barely changes with *x*. In the insulating compound in the vicinity of the Ru-doping-driven insulator–metal transition (*x* = 0.34), the optical excitation across the *J*_eff_ = 1/2 Mott gap is suppressed, and the spectral weight is shifted to lower energies forming a peak structure at about 0.2 eV. A slight increase in *x* from 0.34 to 0.42 leads to a drastic change in the optical spectra. A strong Drude-like peak is registered in the optical conductivity spectra of the compounds with *x* ≥ 0.42. The extended Drude model analysis of the metallic compounds reveals their strange metallic character, suggesting the persistence of the electronic correlations in the metallic compounds. The evolution of the ground state with Ru substitution registered in optical data can be explained in terms of a percolative insulator–metal transition as revealed in a recent STS measurement^22^. The doping evolution of the infrared-active phonon modes displays an intriguing anomaly. While the phonon modes of the insulating and the fully metallic compounds show almost symmetric or weakly asymmetric lineshapes, those of the *x* = 0.34 compound display a pronounced lineshape asymmetry. The temperature evolution of the phonon modes of the *x* = 0.34 compound reveals that the asymmetry is enhanced in the antiferromagnetic state. We discuss relationship among the *J*_eff_ = 1/2 pseudospin of the Ir^4+^ ions, the impurity *S* = 1 spin of the Ru^4+^ ions, and charge excitations for the strong asymmetry of the phonon modes of the *x* = 0.34 compound.

## Results and discussion

### Doping evolution of the electronic response

**Figure 1:**
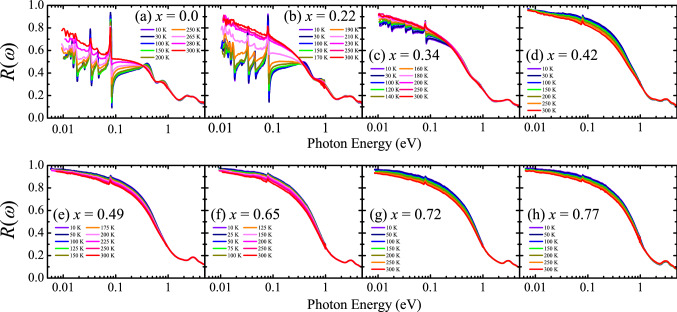
Temperature-dependent reflectivity spectra *R*(*ω*) of Sr_3_(Ir_1-*x*_Ru_*x*_)_2_O_7_ with (**a**) *x* = 0.0, (**b**) *x* = 0.22, (**c**) *x* = 0.34, (**d**) *x* = 0.42, (**e**) *x* = 0.49, (**f**) *x* = 0.65, (**g**) *x* = 0.72, and (**h**) *x* = 0.77 at selected temperatures.

The reflectivity *R*(*ω*) spectra of the Sr_3_(Ir_1-*x*_Ru_x_)_2_O_7_ crystals are displayed in Fig. 1. The ground state of the *x* = 0.0, 0.22, and 0.34 crystals is an antiferromagnetic insulator. The *x* = 0.42, 0.49, 0.65 and 0.72, 0.77 samples have antiferromagnetic metallic and paramagnetic metallic ground states, respectively. The doping-induced insulator–metal transition is known to occur at the critical Ru concentration of *x*_c_ = 0.35^22^. The impact of the insulator–metal transition is clearly seen the *R*(*ω*) data. The low-energy *R*(*ω*) spectra of the *x* = 0.0 and 0.22 compounds shows several sharp features corresponding to infrared-active phonon modes. Upon increasing *x*, the sharp features become progressively weaker. In the fully metallic compounds, i.e., *x* ≥ 0.42, the *R*(*ω*) increases with decreasing energy at all measurement temperatures and the overall magnitude of the *R*(*ω*) are larger than those of the insulating ones, which is due to the response from itinerant carriers in the metallic compounds.

Another notable difference between the *R*(*ω*) data of the insulating and metallic compounds is found in their temperature dependence. The magnitude of the low-energy *R*(*ω*) spectra of the former show a strong decrease with decreasing temperature, which is associated with the band shift driven by antiferromagnetic order^15^. In contrast, the overall level of the low-energy *R*(*ω*) spectra of the metallic compounds increases with decreasing temperature due to the suppression of the scattering of the itinerant carriers.

**Figure 2:**
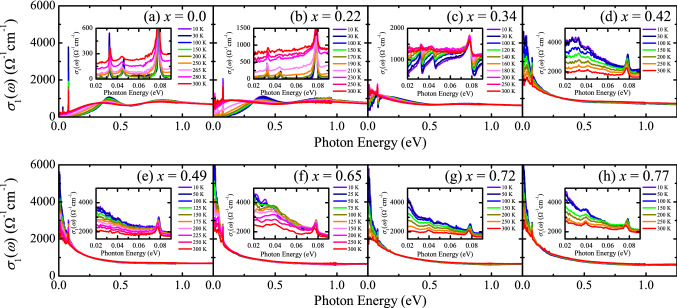
Real part of the optical conductivity spectra *σ*_1_(*ω*) of Sr_3_(Ir_1-*x*_Ru_*x*_)_2_O_7_ with (**a**) *x* = 0.0, (**b**) *x* = 0.22, (**c**) *x* = 0.34, (**d**) *x* = 0.42, (**e**) *x* = 0.49, (**f**) *x* = 0.65, (**g**) *x* = 0.72, and (**h**) *x* = 0.77 at selected temperatures. Insets show *σ*_1_(*ω*) in the energy region below 0.1 eV.

The real part of the optical conductivity *σ*_1_(*ω*) spectra obtained from the Kramers–Kronig analysis^24^ of the *R*(*ω*) data are shown in Fig. 2. The two-peak structure, which is the infrared characteristic of the *J*_eff_ = 1/2 Mott state^6,14^, is clearly seen in *σ*_1_(*ω*) at 10 K of the *x* = 0.0 and 0.22 compounds (Fig. 2a,b). In the *x* = 0.34 compound which shows a thermally driven insulator-to-metal transition at *T*_MIT_ ≈ 135 K and an antiferromagnetic-to-paramagnetic transition at *T*_AF_ ≈ 200 K^22^, the *σ*_1_(*ω*) data at 10 K show a peak at about 0.2 eV which transforms into a Drude-like peak with increasing the temperature. This temperature-dependent change is related to the shift of the bands toward the Fermi level and the resulting appearance of the Fermi surface with the suppression of the antiferromagnetic order^15^. In the metallic compounds (Fig. 2d–h), a Drude-like peak is observed and becomes broader with increasing the temperature.

**Figure 3:**
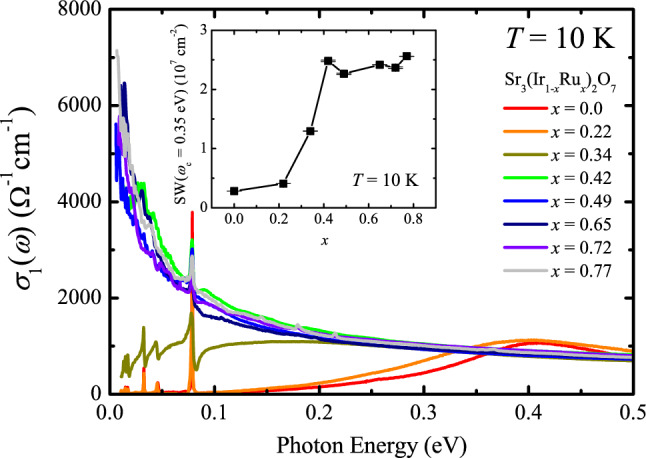
* σ*_1_(*ω*) of Sr_3_(Ir_1-*x*_Ru_*x*_)_2_O_7_ at 10 K in the energy region between 0 and 0.5 eV. Inset shows the spectral weight of the Sr_3_(Ir_1-*x*_Ru_*x*_)_2_O_7_ compounds obtained by integrating *σ*_1_(*ω*) up to 0.35 eV.

In order to identify the evolution of the ground state more clearly, we plot the *σ*_1_(*ω*) data at 10 K of the Sr_3_(Ir_1-*x*_Ru_*x*_)_2_O_7_ compounds in Fig. 3. One can immediately notice that the change in *σ*_1_(*ω*) occurs mainly in the *x* region close to *x*_c_ = 0.35, where the transport data display a Ru-doping-driven insulator–metal transition^22^. The *σ*_1_(*ω*) spectrum of the *x* = 0.22 compound is almost the same as that of the parent compound. Ru substitution is expected to dope holes into the system. The little change in *σ*_1_(*ω*) even upon 22% hole doping is in sharp contrast to the behavior of *σ*_1_(*ω*) of the electron-doped compounds. The electron doping via about 5% substitution of La^3+^ ions in (Sr_1-*y*_La_*y*_)_3_Ir_2_O_7_ leads to a collapse of the Mott gap^16,25^ and the emergence of a Drude-like peak^26^. Our *σ*_1_(*ω*) data suggest that the doped holes in Sr_3_(Ir_1-*x*_Ru_*x*_)_2_O_7_ are localized and the Mott gap remains intact in the *x* = 0.22 compound. With further increasing *x* up to 34% which is very close *x*_c_, the *σ*_1_(*ω*) data display sizeable changes. The optical transition at about 0.4 eV, corresponding to the optical transition between the *J*_eff_ = 1/2 Hubbard bands is suppressed and the spectral weight is shifted to lower energies to form a peak at about 0.2 eV. The spectral weight shift and the large decrease in the optical gap suggest that the compound is on the verge of the insulator–metal transition. Indeed, a small increase in the Ru concentration by 8% yields a drastic change in *σ*_1_(*ω*). A strong Drude-like peak centered at zero energy is observed in the *σ*_1_(*ω*) of the *x* ≥ 0.42 compound. The doping dependence of the low-energy spectral weight (SW) obtained by integrating *σ*_1_(*ω*) up to an isosbetic point at *ω*_c_ = 0.35 eV, displayed in the inset of Fig. 3, clearly reveals that the abrupt insulator–metal transition occurs with increasing *x* across *x*_c_ = 0.35 which is close to the classical two-dimensional percolation threshold of 0.41^22,27^.

**Figure 4:**
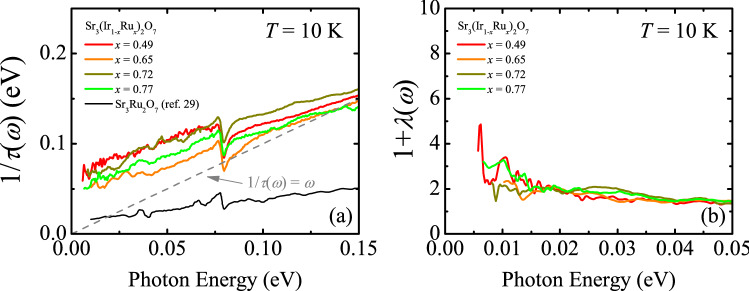
Frequency-dependent (**a**) scattering rate 1/*τ*(*ω*) and (**b**) mass enhancement 1 +*λ* (*ω*) of the metallic Sr_3_(Ir_1-*x*_Ru_*x*_)_2_O_7_ compound at 10 K. The scattering rate spectrum of Sr_3_Ru_2_O_7_ (ref. 29) is plotted in (**a**) for comparison. The dashed line in (**a**) represents 1/* τ* (* ω*) = * ω*.

We carry out the extended Drude model analysis of the optical spectra of the metallic compounds to gain insights into the dynamics of the doped carriers^2^: $\frac{1}{\tau (\omega )}=\frac{{\omega }_{p}^{2}}{4\pi }\mathrm{Re}\left(\frac{1}{\sigma (\omega )}\right)$ and $1+\lambda \left(\omega \right)=-\frac{{\omega }_{p}^{2}}{4\pi \omega }\mathrm{Im}\left(\frac{1}{\sigma (\omega )}\right)$. 1/*τ*(*ω*) and 1 + *λ*(*ω*) is the frequency-dependent scattering rate and the mass enhancement, respectively. *ω*_p_ is the plasma frequency and is obtained by integrating *σ*_1_(*ω*) up to 0.35 eV: ${\omega }_{p}^{2}=8\times {\int }_{0}^{0.35 \mathrm{eV}}{\sigma }_{1}\left(\omega \right)d\omega$. The results of the extended Drude model analysis of the data at 10 K are shown in Fig. 4. For a simple Drude peak, the scattering rate is independent of energy and the mass enhancement is unity^24^. In contrast the scattering rate and the mass enhancement data of Sr_3_(Ir_1-*x*_Ru_*x*_)_2_O_7_ show a clear energy dependence, demonstrating the effects of the electronic correlations. We note that the scattering rate is linear in energy, which is distinct from the *ω*^2^ dependence in a Fermi-liquid metal. Such a linear energy dependence of the scattering rate has been observed in the cuprate superconductors and is linked to their strange metallic phase in which the resistivity exhibits a linear temperature dependence^1^. The resistivity data of the Sr_3_(Ir_1-*x*_Ru_*x*_)_2_O_7_ compounds with *x* ≥ 0.50 also show a linear temperature dependence over a wide range of temperature^22,28^. In addition, we find that the magnitude of the scattering rate is larger than the energy: 1/*τ* (*ω*) > *ω*. The dashed line in Fig. 4a represents 1/*τ* (*ω*) = *ω*. The region below this line corresponds to the Fermi-liquid regime, where the quasiparticles are well defined^1^. The scattering rate data therefore suggest that the intraband response of the metallic Sr_3_(Ir_1-*x*_Ru_*x*_)_2_O_7_ compounds is strongly dissipative. The frequency dependent scattering rate of Sr_3_Ru_2_O_7_ at *T* = 12 K is also plotted in Fig. 4a for comparison^29^. While the scattering rate of Sr_3_Ru_2_O_7_ is also linear in energy, its magnitude is much smaller than those of the metallic Sr_3_(Ir_1-*x*_Ru_*x*_)_2_O_7_ compounds, falling into the Landau Fermi-liquid regime of 1/*τ*(*ω*) < *ω*. Disorder which is inevitably introduced by the Ru substitution can increase the absolute magnitude of the scattering rate of the Sr_3_(Ir_1-*x*_Ru_*x*_)_2_O_7_ compounds, because the disorder can enhance the impurity scattering rate which is frequency independent, thereby resulting in a vertical shift of the 1/*τ*(*ω*) spectra. Nevertheless, we find that the slope of the scattering rate of Sr_3_(Ir_1-*x*_Ru_*x*_)_2_O_7_ is found to be larger than that of Sr_3_Ru_2_O_7_. The value of the mass enhancement of Sr_3_(Ir_1-*x*_Ru_*x*_)_2_O_7_ reaches about 4 at the lowest energy, which is comparable to those of the Ruddlesden-Popper series ruthenates^30–32^. The results of the extended Drude model analysis therefore indicate that the electronic correlations persist even in the metallic compounds and play crucial roles for their charge dynamics.

The doping evolution of the electronic response revealed in our optical data provides a global picture of the insulator–metal transition of the Sr_3_(Ir_1-*x*_Ru_*x*_)_2_O_7_ system. The localization of doped holes at low Ru concentrations and the drastic changes in the electronic structure with the increase in *x* near the classical two-dimensional percolation threshold support a percolative insulator–metal transition, which is indeed revealed in a STS measurement^22^. The strong dissipation of the conduction in the metallic compounds reveals that the electronic correlations in conjunction with disorder play an important role for the dynamics of doped charge carriers.

**Figure 5:**
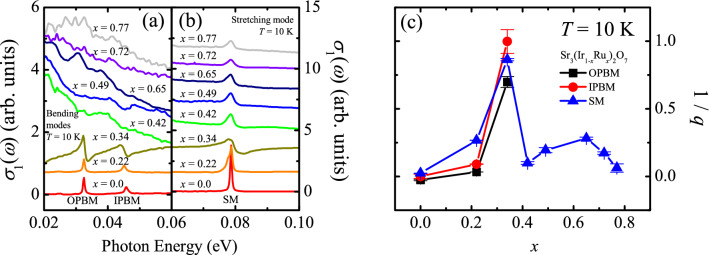
Low-energy *σ*_1_(*ω*) of Sr_3_(Ir_1-*x*_Ru_*x*_)_2_O_7_ at 10 K. (**a**) The out-of-plane bending mode (OPBM) at 33 meV and the in-plane bending mode (IPBM) at 45 meV are shown. (**b**) The stretching mode (SM) at 78 meV is shown. The baseline of each spectrum is shifted for clarity. (**c**) Doping dependence of the inverse of the Fano parameter, i.e., 1/*q*.

The insulator–metal transition of the Sr_3_(Ir_1-*x*_Ru_*x*_)_2_O_7_ system should be contrasted to that of *B*-site-substituted single-layer cousins. The antiferromagnetic-insulator-to-paramagnetic-metal transition in Sr_2_(Ir,Ru)O_4_ was attributed to the structural phase transition from a *I*4_1_/*acd* to *I*4/*mmm* tetragonal structure accompanying abrupt decrease in the *a*- and *c*-axes lattice constants^33,34^. This structural change can result in the increase in the electronic bandwidth. Our X-ray and neutron diffraction data reveal that the lattice parameters of the Sr_3_(Ir_1-*x*_Ru_*x*_)_2_O_7_ system changes gradually without any anomaly due to structural transitions^22,28^. We also remark that the phonon spectra shown in Fig. 5 do not show any splitting and/or appearance of new modes, further suggesting the absence of the structural transition with Ru substitution.

In Sr_2_(Ir,Rh)O_4_ of which end members share the same *I*4_1_/*acd* structure, there have been discussion on the origin of insulator–metal transition. It is suggested that Rh doping results in an isoelectronic substitution of Ir^4+^ (5*d*^5^) ions for Rh^4+^ (4*d*^5^) ions and that the insulator–metal transition is driven by a tuning of the effective spin–orbit coupling^35^. Another study suggests that the insulator–metal transition is associated with the emergence of impurity bands of Rh character and its overlap with lower Hubbard band^36^ while the doping is isoelectronic. On the other hand, other studies report that the spin–orbit coupling is robust against Rh doping^36,37^ and Rh is substituted as Rh^3+^ (4*d*^6^), leading to hole doping into the Ir sites^37–39^. In the Sr_3_(Ir_1-x_Ru_*x*_)_2_O_7_ crystals, an X-ray absorption spectroscopy shows that the charge disproportionation does not occur^23^. STS studies of Sr_3_(Ir_1-x_Ru_x_)_2_O_7_ further show that impurity bands do not form upon Ru doping and that the insulator–metal transition is a percolation type^22,23^.

### Doping evolution of phonon dynamics

We now focus on the phonon dynamics of the Sr_3_(Ir_1-*x*_Ru_*x*_)_2_O_7_ system. Figure 5a,b show the *σ*_1_(*ω*) spectra of the Sr_3_(Ir_1-*x*_Ru_*x*_)_2_O_7_ compounds at 10 K in the far-infrared region where the peaks due to the Ir–O–Ir bond bending modes (33 meV, 45 meV) and Ir–O bond stretching mode (78 meV) are observed, respectively^14,40^. In general, introduction of charge carriers induces the screening of the polarization induced by the phonon modes, leading to the suppression of the phonon peaks in the optical spectra. However, the phonon modes remain robust against 22% substitution of Ru ions, which again indicates that the doped holes are localized. Upon further Ru substitution, the phonon modes weaken drastically. In the fully metallic compounds, the bending modes are hardly seen and only the stretching mode is observed.

The most notable finding from the phonon data at 10 K is the pronounced asymmetry in the lineshapes of the phonon modes of the *x* = 0.34 compound. At low dopings of *x* = 0.0 and 0.22, the phonons appear to have almost symmetric lineshape. With the appearance of charge excitations represented by the finite low-energy conductivity of the *x* = 0.34 compound, the phonon modes show the conspicuous asymmetry. In the fully metallic compounds, *x* ≥ 0.42, the asymmetry in the lineshape of the stretching modes is observed but the degree of the asymmetry is much weaker than that of the *x* = 0.34 compound.

The coupling between a sharp mode and a broad continuum due to excitations of charge or spin degrees of freedom can result in asymmetric phonon peaks which can be modeled by Fano-type oscillator model^41–43^: ${\sigma }_{1}\left(\omega \right)=\frac{{\omega }_{p}^{2}}{4\pi \gamma }\frac{{q}^{2}+2qx-1}{{q}^{2}(1+{x}^{2})}$ with $x=2(\omega -{\omega }_{0})/\gamma$. Here *ω*_0_, *ω*_p_, γ, and *q* are the resonance energy, the plasma frequency, the linewidth, and the Fano asymmetry parameter of the phonon modes, respectively. The inverse of the Fano asymmetry parameter, 1/*q,* represents the strength of the coupling and quantifies the degree of the asymmetry. When |1/*q*| $\ll$ 1, the Fano oscillator approaches the Lorentz oscillator.

In order to gain insights into the origin of the pronounced asymmetry of the *x* = 0.34 compound, we fit the conductivity data at 10 K of all the compounds. The electronic background is fitted by using a combination of the Drude and Lorentz oscillators, and the phonon peaks are fitted by using Fano-type oscillators. The bending/stretching modes and the stretching mode are included in the fitting for *x* ≤ 0.34 and for *x* ≥ 0.42, respectively. The *x* dependence of the asymmetry parameter 1/*q* extracted from the fit is shown in Fig. 5c. In the parent compound, 1/*q* is essentially zero and their phonon peaks can be fitted by the Lorentz oscillator. While 22% Ru substitution induces an increase in 1/*q*, the drastic increase is observed in the *x* = 0.34 compound. We note this large increase coincides with the onset of the incoherent charge excitations in the *x* = 0.34 compound, represented by rather flat shape of the optical conductivity in the inset of Fig. 2c. However, it decreases to smaller values despite the observation that the low-energy spectral weight from the doped charge carriers is enhanced at *x* ≥ 0.42. These results suggest a complex interplay between the phonons, charge excitations, and antiferromagnetic order in the Sr_3_(Ir_1-*x*_Ru_*x*_)_2_O_7_ system.

**Figure 6:**
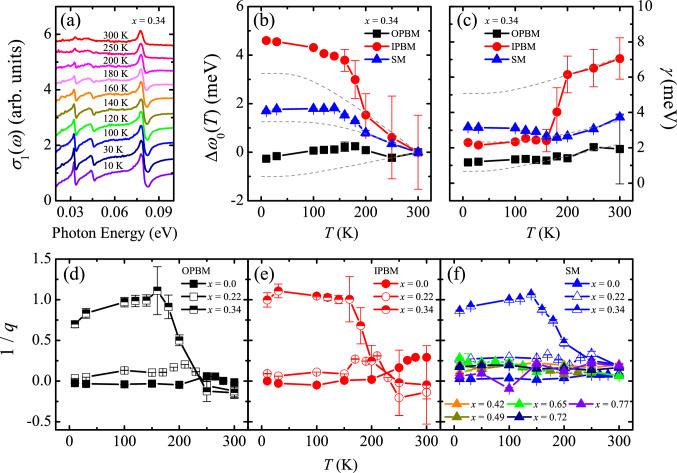
(**a**) Temperature dependent *σ*_1_(*ω*) of the *x* = 0.34 compound in the energy region between 0.02 and 0.1 eV. The baseline of each spectrum is shifted for clarity. Temperature dependence of (**b**) the resonance energies and of (**c**) the linewidths of the phonon modes of the *x* = 0.34 compound. The dashed lines in (**b**,**c**) correspond to the theoretical prediction due to phonon–phonon anharmonic interactions. Temperature dependence of 1/*q* of the out-of-plane bending mode (OPBM), (**e**) the in-plane bending mode (IPBM), and (**f**) the stretching mode (SM) of the Sr_3_(Ir_1-*x*_Ru_*x*_)_2_O_7_ compounds.

Temperature dependence of the phonon modes of the *x* = 0.34 compound reveals phonon anomalies due to pseudospin-phonon coupling. Figure 6a displays the temperature dependence of the phonon modes of the *x* = 0.34 compound. The spectra are shifted for clarity. The *x* = 0.34 compound exhibits a paramagnetic-to-antiferromagnetic transition with decreasing the temperature across *T*_AF_ $\approx$ 200 K^22^. As shown in Fig. 6b,c, the resonance energies and the linewidths of the phonon modes display anomalies at *T*_AF_, which cannot be described by the anharmonic phonon–phonon interactions that lead to gradual hardening and narrowing of the phonon peaks with decreasing the temperature^44^ (dashed lines in Fig. 6b,c). Such anomalies are not observed in the infrared-active phonon modes of Sr_3_Ir_2_O_7_^45^, suggesting a strong pseudospin-phonon coupling in the *x* = 0.34 compound. We also find that the asymmetry parameter increases drastically in the antiferromagnetic state (Fig. 6d–f).

Having inferred the importance of the pseudospin-phonon coupling, we discuss why the pseudospin-phonon coupling is particularly effective in the *x* = 0.34 compound for inducing the phonon asymmetry, although the Sr_3_(Ir_1-*x*_Ru_*x*_)_2_O_7_ with 0.0 ≤ *x* < 0.70 are antiferromagnetic^22^. A combination of the results of neutron scattering^22^ and our optical measurements provides a conjecture on this phenomenon. The neutron scattering experiments on Sr_3_(Ir_1-*x*_Ru_*x*_)_2_O_7_ show an anomalous enhancement of the antiferromagnetic order parameter at *x* = 0.33^22^. Excluding the data point for the *x* = 0.33 compounds, Ru substitution induces a rapid and linear suppression of the antiferromagnetic order parameter; the antiferromagnetic order parameter decreases down to the value of about 20% of that of the parent compound with 50% Ru substitution due to the dilution of ordered Ir^4+^ ions by localized Ru^4+^ *S* = 1 impurities in the lightly Ru-doped regime and due to the screening by itinerant carriers in the fully metallic regime^22^. However, the order parameter of the *x* = 0.33 compound deviates from the overall linear trend in the *x* dependence and has anomalously large value which is nearly the same as that of the parent compound. This anomaly is attributed to possible induced ordering of *S* = 1 moments from doped Ru^4+^ (4*d*^4^) ions^22^. We note that the interface density between Ir and Ru rich regions or ordered and disordered moments is in principle maximum at the percolation threshold, which might play a critical role for the enhanced antiferromagnetic order and the phonon anomalies of the *x* = 0.34 compound.

The pseudospin-phonon coupling is manifested in previous spectroscopic studies on layered iridates. Raman^46,47^ and ultrafast^48^ spectroscopy studies of Sr_2_IrO_4_ and Sr_3_Ir_2_O_7_ show anomalies of the frequencies and the linewidths of phonon modes at *T*_AF_, which is similar with our observation summarized in Fig. 6b,c. However, the temperature evolution of the lineshape asymmetry of the *x* = 0.34 compound is in contrast to those of the Raman-active phonon modes of Sr_2_IrO_4_ and Sr_3_Ir_2_O_7_^47^. In the Raman spectra, the phonon mode which modulates the in-plane Ir–O–Ir bond is almost symmetric in the antiferromagnetic state and becomes asymmetric with |1/*q*| $\ll$ 0.2 as the temperature increases across *T*_AF_, which is ascribed to the fluctuation of the *J*_eff_ = 1/2 pseudospins^47^. As shown in Fig. 6e, the asymmetry parameter of the in-plane bending mode (IPBM) in our *σ*_1_(*ω*) data of Sr_3_Ir_2_O_7_ displays similar temperature evolution. In contrast, the asymmetry of the phonon modes of the *x* = 0.34 compound is enhanced in the antiferromagnetic state (Fig. 6d–f). In addition, the magnitude of the asymmetry parameter is much larger than the values from the Raman studies^47,49^ as well as the values of the phonon modes of the other Sr_3_(Ir_1-*x*_Ru_*x*_)_2_O_7_ compounds. What distinguish the *x* = 0.34 compound from the parent and the other Ru-doped compounds are the presence of the low-energy incoherent charge excitations at low temperatures (Fig. 2) and the larger antiferromagnetic order parameter^22^, respectively. This distinction suggests the importance of the *S* = 1 impurity spins of Ru^4+^ ions and the incoherent charge excitations for the asymmetry of the phonons of the *x* = 0.34 sample. Indeed, La doping into Sr_2_IrO_4_ is found to enhance the phonon asymmetry^49^. Studies on phonon dynamics of the parent compound at high pressures^50^ which can induce low-energy charge excitations or of Sr_2_(Ir,Ru)O_4_ compounds^51^ located in the vicinity of the insulator–metal transition may provide further information on the coupling among phonon, charge excitations, and antiferromagnetism.

## Conclusion

We investigated the electronic response and the phonon dynamics of the Sr_3_(Ir_1-*x*_Ru_*x*_)_2_O_7_ system by using optical spectroscopy. We find that the Ru doping induces the drastic changes in the electronic structure when the Ru concentration increases across the critical value *x*_c_ = 0.35 at which the insulator–metal transition takes place. At low dopings, the *J*_eff_ = 1/2 Mott state remains robust against Ru substitution, indicating that the doped holes are localized. In the insulating side in close vicinity of the insulator–metal transition boundary, a slight increase in the Ru concentration leads to drastic changes in the electronic structure, which is associated with the collapse of the Mott state and concomitant emergence of the Drude-like response from the itinerant carriers. Such observations support that the Ru-doping-driven insulator–metal transition may have a percolative nature, which is consistent with the observation from a STS study^22^. The extended Drude model analysis reveals a persistence of the electronic correlations in the fully metallic compounds. The far-infrared response reveals intriguing manifestations of the pseudospin-phonon coupling. While the phonon modes of the insulating and the metallic compounds have nearly symmetric lineshapes, the phonon peaks of the antiferromagnetic and barely insulating compound (*x* = 0.34) show a pronounced asymmetry. The temperature evolutions of the resonance energy, the linewidth, and the degree of asymmetry display distinct anomalies at *T*_AF_, indicating a pseudospin-phonon coupling. Our data in conjunction with recent neutron scattering, magnetization, and STS studies^22,23^ suggest an importance of the impurity *S* = 1 spins of Ru ions and incoherent charge excitations for the strong lineshape asymmetry of phonon modes of the *x* = 0.34 compound.

## Methods

High-quality single-crystals of Sr_3_(Ir_1-*x*_Ru_*x*_)_2_O_7_ (*x* = 0.0, 0.22, 0.34, 0.42, 0.49, 0.65, 0.72, and 0.77) were grown via flux techniques. Dopant content was determined by energy-dispersive X-ray spectroscopy measurements which show a homogeneous Ru distribution within a central value ± 3% (Fig. S2 and Table S1 of the Supplemental Material). The X-ray diffraction measurements reveal no impurity phases within instrument resolution (~ 2–3%) (Fig. S3 of the Supplemental Material). Details of the growth procedure and characterizations were also described elsewhere^22,23,28^.

We measured the *ab*-plane reflectivity spectra *R*(*ω*) in the photon energy region between 5 meV and 1 eV using a Fourier transform infrared spectrometer (VERTEX 70v, Bruker) with the *in-situ* gold overcoating technique^52^. We employed spectroscopic ellipsometer (V-VASE and M-2000, J. A. Woollam Co.) to obtain the complex optical conductivity, *σ* (* ω*) = * σ*_1_(*ω*) + *iσ*_2_(*ω*), in the energy range from 0.74 to 5 eV. For the low-energy spectra below 5 meV, *R*(*ω*) was extrapolated by using the Hagen-Rubens relation^24^. We carried out the Kramers–Kronig analysis of the *R*(*ω*) to obtain *σ* (*ω*).

## Supplementary Information

Supplementary Information

## References

[1] Basov DN, Timusk T (2005). Electrodynamics of high-*T*_*c*_ superconductors. Rev. Mod. Phys..

[2] Basov DN, Averitt RD, van der Marel D, Dressel M, Haule K (2011). Electrodynamics of correlated electron materials. Rev. Mod. Phys..

[3] Keimer B, Kivelson SA, Norman MR, Uchida S, Zaanen J (2015). From quantum matter to high-temperature superconductivity in copper oxides. Nature.

[4] Kim BJ (2008). Novel *J*_eff_ =1/2 mott state induced by relativistic spin–orbit coupling in Sr_2_IrO_4_. Phys. Rev. Lett..

[5] Kim BJ (2009). Phase-sensitive observation of a spin-orbital mott state in Sr_2_IrO_4_. Science.

[6] Moon SJ (2008). Dimensionality-controlled insulator-metal transition and correlated metallic state in 5*d* transition metal oxides Sr_*n*__+1_Ir_*n*_O_3__*n*__+1_ (*n* = 1, 2, and ∞). Phys. Rev. Lett..

[7] Jin H, Jeong H, Ozaki T, Yu J (2009). Anisotropic exchange interactions of spin-orbit-integrated states in Sr_2_IrO_4_. Phys. Rev. B.

[8] Kim YK (2014). Fermi arcs in a doped pseudospin-1/2 Heisenberg antiferromagnet. Science.

[9] de la Torre A (2015). Collapse of the mott gap and emergence of a nodal liquid in lightly doped Sr_2_IrO_4_. Phys. Rev. Lett..

[10] Kim YK, Sung NH, Denlinger JD, Kim BJ (2016). Observation of a *d*-wave gap in electron-doped Sr_2_IrO_4_. Nat. Phys..

[11] Battisti I (2017). Universality of pseudogap and emergent order in lightly doped Mott insulators. Nat. Phys..

[12] Yan YJ (2015). Electron-doped Sr_2_IrO_4_: An analogue of hole-doped cuprate superconductors demonstrated by scanning tunneling microscopy. Phys. Rev. X.

[13] Chen X (2018). Unidirectional spin density wave state in metallic (Sr_1-__*x*_La_*x*_)_2_IrO_4_. Nat. Commun..

[14] Moon SJ (2009). Temperature dependence of the elctronic structure of the *J*_eff_ = 1/2 Mott insulator Sr_2_IrO_4_ studied by optical spectroscopy. Phys. Rev. B.

[15] Song S (2018). Magnetically driven band shift and metal-insulator transition in spin-orbit-coupled Sr_3_(Ir_1-__*x*_Ru_*x*_)_2_O_7_. Phys. Rev. B.

[16] Hogan T (2015). First-order melting of a weak spin-orbit mott insulator into a correlated metal. Phys. Rev. Lett..

[17] Chen X (2015). Influence of electron doping on the ground state of (Sr_1-__*x*_La_*x*_)_2_IrO_4_. Phys. Rev. B.

[18] Wang Z (2019). Doping induced Mott collapse and possible density wave instabilities in (Sr_1-__*x*_La_*x*_)_3_Ir_2_O_7_. NPJ Quant. Mater..

[19] Jin W (2019). Polarized Raman spectroscopy study of metallic (Sr_1-__*x*_La_*x*_)_3_Ir_2_O_7_: A consistent picture of disorder-interrupted unidirectional charge order. Phys. Rev. B.

[20] Hogan T, Wang X, Chu H, Hsieh D, Wilson SD (2017). Doping-driven structural distortion in the bilayer iridate (Sr_1-__*x*_La_*x*_)_3_Ir_2_O_7_. Phys. Rev. B.

[21] Chu H (2017). A charge density wave-like instability in a doped spin–orbit-assisted weak Mott insulator. Nat. Mater..

[22] Dhital C (2014). Carrier localization and electronic phase separation in a doped spin-orbit-driven Mott phase in Sr_3_(Ir_1-__*x*_Ru_*x*_)_2_O_7_. Nat. Commun..

[23] Wang Z (2018). Disorder induced power-law gaps in an insulator–metal Mott transition. Proc. Natl. Acad. Sci. USA.

[24] Dressel M, Grüner G (2002). Electrodynamics of Solids.

[25] de la Torre A (2014). Coherent quasiparticles with a small fermi surface in lightly doped Sr_3_Ir_2_O_7_. Phys. Rev. Lett..

[26] Ahn G, Song SJ, Hogan T, Wilson SD, Moon SJ (2016). Infrared spectroscopic evidences of strong electronic correlations in (Sr_1-__*x*_La_*x*_)_3_Ir_2_O_7_. Sci. Rep..

[27] Shante VKS, Kirkpatrick S (1971). An introduction to percolation theory. Adv. Phys..

[28] Schmehr JL (2019). Overdamped antiferromagnetic strange metal state in Sr_3_IrRuO_7_. Phys. Rev. Lett..

[29] Mirri C (2008). Anisotropic optical conductivity of Sr_3_Ru_2_O_7_. Phys. Rev. B.

[30] Kostic P (1998). Non-fermi-liquid behavior of SrRuO_3_: Evidence from infrared conductivity. Phys. Rev. Lett..

[31] Katsufuji T, Kasai M, Tokura Y (1996). In-plane and out-of-plane optical spectra of Sr_2_RuO_4_. Phys. Rev. Lett..

[32] Mirri C (2012). Anisotropic optical conductivity of Sr_4_Ru_3_O_10_. Phys. Rev. B.

[33] Cava RJ (1994). Localized-to-itinerant electron transition in Sr_2_Ir_1-__*x*_Ru_*x*_O_4_. Phys. Rev. B.

[34] Yuan SJ (2015). From *J*_eff_ = 1/2 insulator to *p*-wave superconductor in single-crystal Sr_2_Ir_1-__*x*_Ru_*x*_O_4_ (0 ≤ *x* ≤ 1). Phys. Rev. B.

[35] Qi TF (2012). Spin-orbit tuned metal-insulator transitions in single-crystal Sr_2_Ir_1-__*x*_Rh_*x*_O_4_ (0 ≤ *x* ≤ 1). Phys. Rev. B.

[36] Chikara S (2015). Sr_2_Ir_1-__*x*_Rh_*x*_O_4_ (*x* < 1): An inhomogeneous *j*_eff_ = 1/2 Hubbard system. Phys. Rev. B.

[37] Clancy JP (2014). Dilute magnetism and spin-orbital percolation effects in Sr_2_Ir_1-__*x*_Rh_*x*_O_4_. Phys. Rev. B.

[38] Cao Y (2016). Hallmarks of the Mott-metal crossover in the hole-doped pseudospin-1/2 Mott insulator Sr_2_IrO_4_. Nat. Commun..

[39] Chikara S (2017). Charge partitioning and anomalous hole doping in Rh-doped Sr_2_IrO_4_. Phys. Rev. B.

[40] Pröpper D (2016). Optical anisotropy of the *J*_eff_ = 1/2 Mott insulator Sr_2_IrO_4_. Phys. Rev. B.

[41] Fano U (1961). Effects of configuration interaction on intensities and phase shifts. Phys. Rev..

[42] Damascelli A, Schulte K, van der Marel D, Menovsky AA (1997). Infrared spectroscopic study of phonons coupled to charge excitations in FeSi. Phys. Rev. B.

[43] Kuzmenko AB (2009). Gate tunable infrared phonon anomalies in bilayer graphene. Phys. Rev. Lett..

[44] Menéndez J, Cardona M (1984). Temperature dependence of the first-order Raman scattering by phonons in Si, Ge, and α-Sn: Anharmonic effects. Phys. Rev. B.

[45] Park HJ (2014). Phonon-assisted optical excitation in the narrow bandgap Mott insulator Sr_3_Ir_2_O_7_. Phys. Rev. B.

[46] Samanta K, Rigitano D, Pagliuso PG, Granado E (2019). Isospin-phonon coupling and Fano-interference in spin-orbit Mott insulator Sr_2_IrO_4_. Appl. Phys. Lett..

[47] Gretarsson H (2016). Two-magnon raman scattering and pseudospin–lattice interactions in Sr_2_IrO_4_ and Sr_3_Ir_2_O_7_. Phys. Rev. Lett..

[48] Hu LL (2019). Strong pseudospin-lattice coupling in Sr_3_Ir_2_O_7_: Coherent phonon anomaly and negative thermal expansion. Phys. Rev. B.

[49] Gretarsson H (2017). Raman scattering study of vibrational and magnetic excitations in Sr_2-__*x*_La_*x*_IrO_4_. Phys. Rev. B.

[50] Wu Y, Yin X, Hasaien J, Ding Y, Zhao J (2020). High-pressure ultrafast dynamics in Sr_2_IrO_4_: Pressure-induced phonon bottleneck effect. Chin. Phys. Lett..

[51] Glamazda A (2014). Effects of hole doping on magnetic and lattice excitations in Sr_2_Ir_1-*x*_Ru*x*O_4_ (*x* = 0–0.2). Phys. Rev. B.

[52] Homes CC, Reedyk M, Cradles DA, Timusk T (1993). Technique for measuring the reflectance of irregular, submillimeter-sized samples. Appl. Opt..

