# Supplementary material for: Doping and temperature evolutions of optical response of Sr3(Ir1-xRux)2O7
Source: Sci Rep. 2020 Dec 18;10:22340. doi: 10.1038/s41598-020-79263-5 (PMC7749133; doi:10.1038/s41598-020-79263-5)
Supplement: Supplementary file 1 — Supplementary Information [file 41598_2020_79263_MOESM1_ESM.pdf]

SUPPLEMENTAL MATERIAL

Doping and Temperature Evolutions of Optical Response of $Sr_3(Ir_{1-x}Ru_x)_2O_7$

Gihyeon Ahn[1], J. L. Schmehr[3], Z. Porter[3], S. D. Wilson[3] & S. J. Moon[1,3*]

[1]*Department of Physics, Hanyang University, Seoul 04763, Republic of Korea*

[2]*Materials Department, University of California, Santa Barbara, California 93106, USA*

[3]*Research Institute of Natural Science, Hanyang University, Seoul 04763, Republic of Korea*

Sample pictures

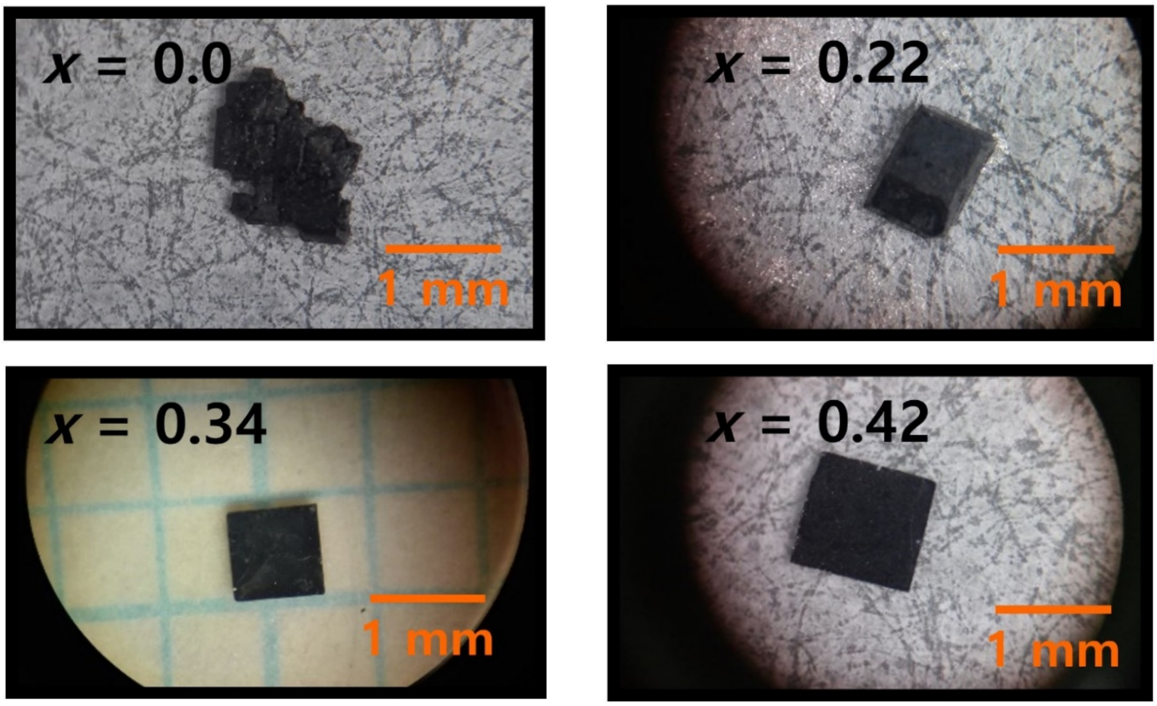

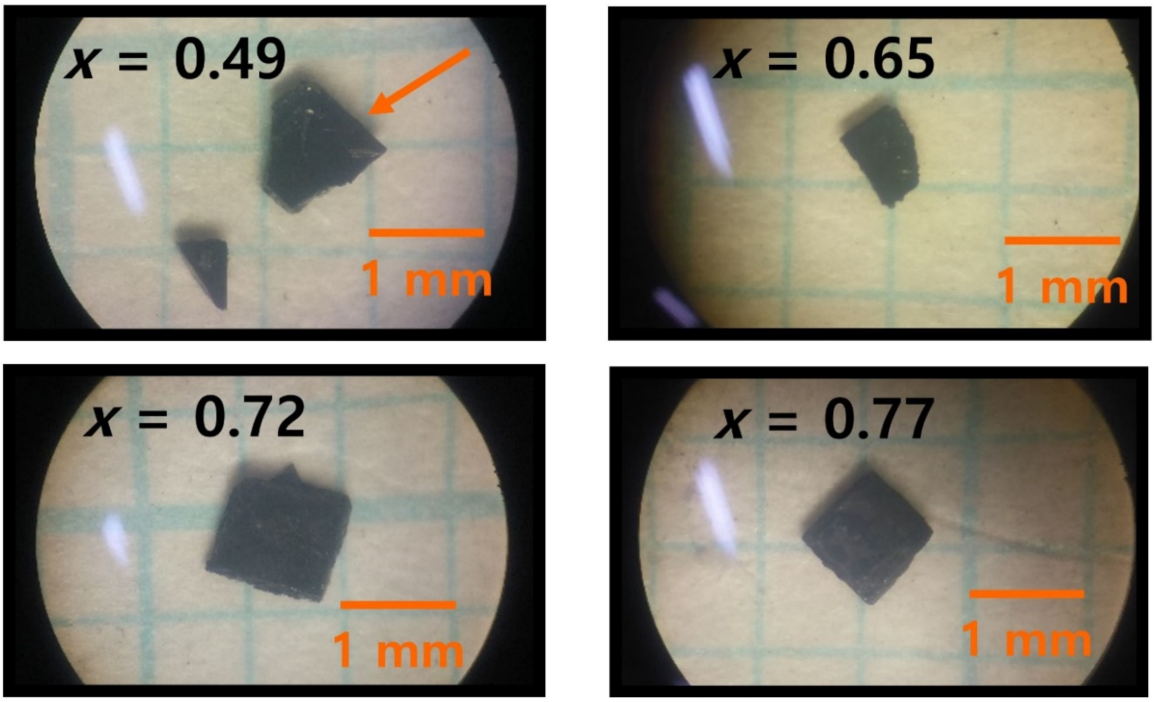

Fig. S1. Pictures of single crystal $Sr_3(Ir_{1-x}Ru_x)_2O_7$ samples taken by using optical microscope. Flat cleaved surfaces show high-quality of the samples.

Energy-dispersive X-ray spectroscopy

Energy-dispersive X-ray spectroscopy (EDS) was measured with a ThermoFisher Apreo C scanning electron microscope equipped with an EDAX Si-drift EDS detector. Cleaved crystal samples were mounted on carbon tape and the electron beam settings were 20 keV and 0.80 nA. We measured EDS spectra for at least 4 points distributed across each sample, and measured for 50 s with 3.84 microseconds amp time so that the detector dead time was nearly 30%. The reported Ru content x is the mean value from the quantification in the EDAX Genesis software, and the uncertainty is the standard deviation of the measurements. The data show a homogeneous Ru distribution within a central value of ±3%.

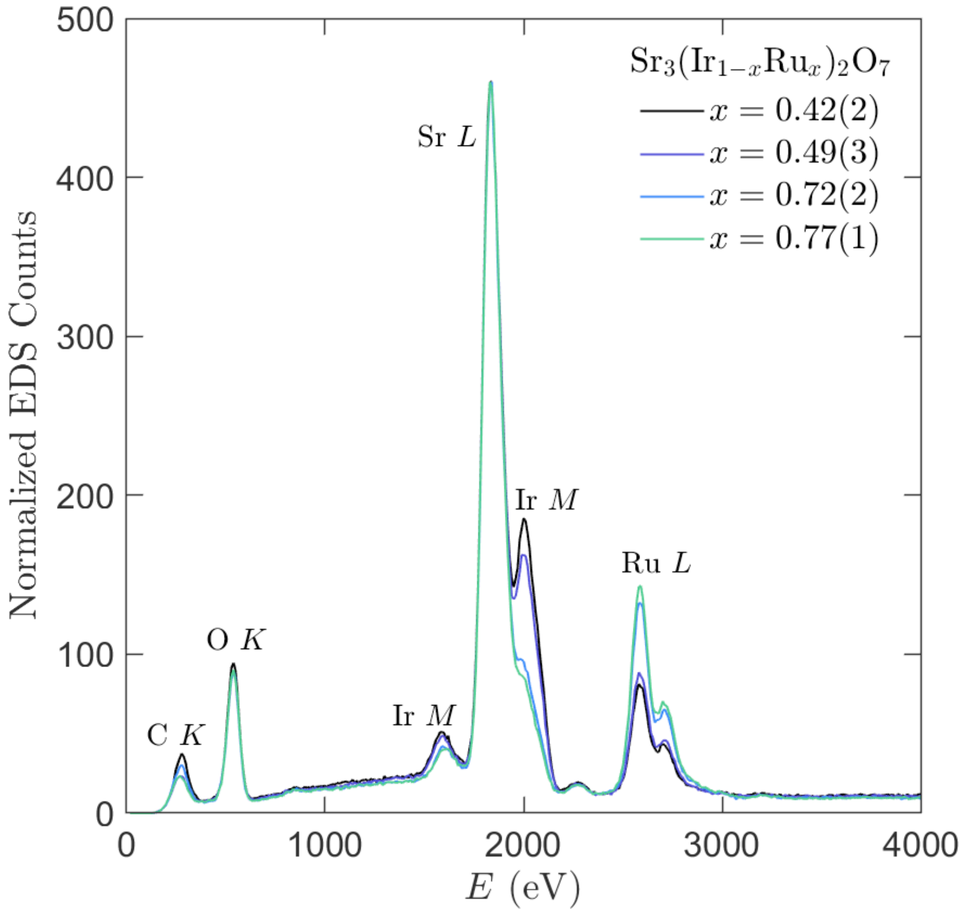

FIG. S2. Representative EDS spectra for four samples. These are histograms with bin size 10 eV, plotted as lines and normalized to the Sr *L* peaks for clarity. Substitution of Ir for Ru is clearly shown by increase (decrease) in Ru *L* (Ir *M*) peak areas as Ru content *x* increases. We note that the C and O *K* peaks are unreliable. Thin carbon/oxygen layers on sample surfaces are results of the exposure to the air.

TABLE S1. EDS values with standard deviations.

Sample	EDS values (%)	Standard deviation (%)
$x = 0.34$	34.3	0.58
$x = 0.42$	42	2
$x = 0.49$	49	3
$x = 0.65$	65.5	1.56
$x = 0.72$	72	2
$x = 0.77$	77	1

X-ray diffraction (XRD) measurement

The samples studied in this paper were checked by a PANalytical Empyrean X-ray diffractometer at room temperature to exclude any possible impurity phases. The samples were placed flat on zero-diffraction plates to measure in the (00l) scattering plane, where impurity peaks such as $Sr_2(Ir_{1-x}Ru_x)O_4$ would be clearly visible. We find no impurity peaks in the XRD spectra within instrument resolution (~2-3 %) as shown in Fig. S3.

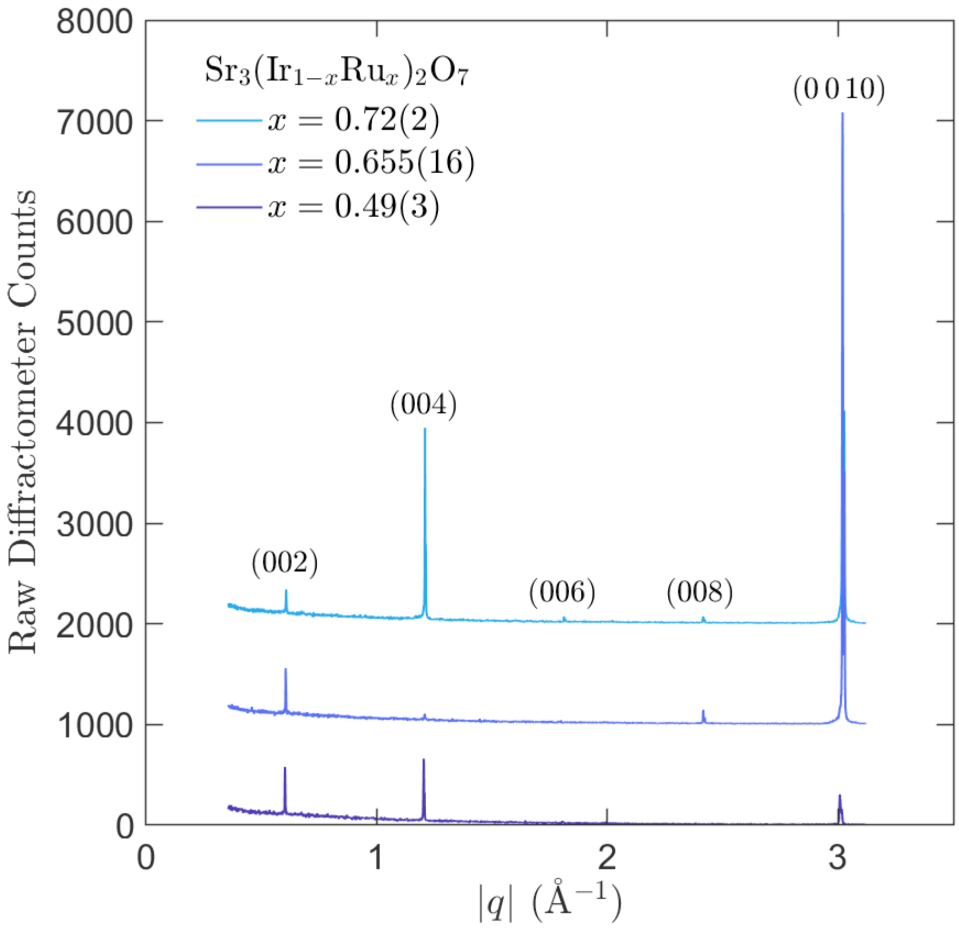

FIG. S3. Representative X-ray diffraction *Q* scans for several samples. The $Sr_3(Ir_{1-x}Ru_x)_2O_7$ peaks are indexed. Note that there are no impurity peaks visible within experimental resolution. The data of the x = 0.72(2) and 0.655(16) samples are vertically shifted for clarity.
